# Supplementary material for: Development of a One-Step Multiplex qPCR Assay for Detection of Methicillin and Vancomycin Drug Resistance Genes in Antibiotic-Resistant Bacteria
Source: Pathogens. 2024 Sep 30;13(10):853. doi: 10.3390/pathogens13100853 (PMC11509969; doi:10.3390/pathogens13100853)
Supplement: Supplementary file 1 [file pathogens-13-00853-s001.zip › pathogens-3218639-supplementary.pdf]

**Supplementary Materials**

**Table S1.** Bacterial reference strains.

| Microorganisms | Antimicrobial susceptibility | Source | Strain designation | Strain characteristics |
|---|---|---|---|---|
| *Staphylococcus aureus* | MRSA | ATCC | ATCC BAA-2094 | *mecA*-positive |
| *Enterococcus faecium* | VRE | NCCP | NCCP 11517 | *vanA*-positive |
| *Enterococcus faecium* | VRE | NCCP | NCCP 11519 | *vanB*-positive |
| *Staphylococcus aureus* | MSSA | ATCC | ATCC 29213 | *mecA*-negative |
| *Enterococcus faecalis* | VSE | KCTC | KCTC 3511 | *vanA* and *vanB*- negative |

Abbreviations: MRSA, methicillin-resistant *Staphylococcus aureus*; MSSA, methicillin-susceptible *S. aureus*; VRE, vancomycin-resistant enterococci; VSE, vancomycin- susceptible enterococci; KCTC, Korean Collection for Type Cultures, Taejon, South Korea; ATCC, American Type Culture Collection; NCCP, National Culture Collection for Pathogens, Cheongju, Chungcheongbuk-do, South Korea

**Table S2.** Accession numbers and gene synthesis sequences of the *mecA* and *vanA*-*vanB* genes.

| Name of genes | Organism | Accession No. | Gene synthesis sequences | Amplicon size |
|---|---|---|---|---|
| *mecA* | MRSA; methicillin-resistant *Staphylococcus aureus* (*mecA* positive) | MN635691.1<br>MN635692.1<br>MN635693.1<br>MN720357.1<br>MN720355.1<br>KM505043.1 | 5'-GGTAATATCGACTTAAAACAAGCAATAGAATCATCAGATAACATTTTCTTTGCTAGAGTAGCACTCGAATTAGGCAGTAAGAAATTTGAAAAAGGCATGAAAAAACTAGGTGTTGGTGAAGATATACCAAGTGATTATCCATTTTATAATGCTCAAATTTCAAACAAAAATTTAGATAATGAAATATTATTAGCTGATTCAGGTTACGGACAGGGTGAAATATT-3' | 224 bp |
| *vanA*-*vanB* | VRE; vancomycin-resistant enterococci (*vanA* positive) | NG_048325.1<br>NG_048323.1<br>MF459049.1<br>KR047792.1 | 5'-ACTTAACGCTGCGATAGAAGCGGCAGGACAATATGATGGAAAAATCTTAATTGAGCAAGCGATTTCGGGCTGTGAGGTCGGTTGTGCGGTATTGGGAAACAGTGCCGCGTTAGCTGTTGGCGAGGTGGACCAAATCAGGCTGCAGTACGGAATCTTTCGTATTCATCAGGAAGTCGAGCCGGAAAAAGGCTCTGAAAATGCGATGATTACAGTTCCCGCAGACATTCCGGTCGAGGAACGAAATCG-3' | 246 bp |
| | VRE; vancomycin-resistant enterococci (*vanB* positive) | NG_048331.1<br>NG_048332.1<br>NG_048333.1<br>NG_048334.1 | | |

**Table S3.** Confirmation for positive control of *mecA* and *vanA-vanB* genes by sequencing.

| Plasmid gene | One-step Multiplex qPCR Assay | Sequencing primer | Accession | Description | Maximum score | Total score | Query cover | E value | Percent Identified |
|---|---|---|---|---|---|---|---|---|---|
| | *mecA* (FAM) and *vanA-vanB* (HEX) | | | | | | | | |
| *mecA* | Positive (FAM) | *mecA* primer | MN720353.1 | *Staphylococcus aureus* strain TMZ112 MecA (*mecA*) gene, partial cds | 278 | 278 | 100% | $4 \times 10^{-70}$ | 98.73 % |
| *vanA-vanB* | Positive (HEX) | *vanA-vanB* primer | MN478489.1 | *Enterococcus faecium* strain ATCC 700221 VanA ligase (*vanA*) gene, partial cds | 270 | 270 | 97% | $8 \times 10^{-68}$ | 94.83 % |

**Table S4.** Results of singleplex, multiplex real-time PCR, and sequencing for reference strains as positive and negative controls in this study.

| Organism | Control type | Singleplex qPCR (Mean Ct± SD) | | Multiplex qPCR (Mean Ct± SD) | Sequencing primer | Accession | Description | Maximum score | Total score | Query cover | E value | Percent Identified |
|---|---|---|---|---|---|---|---|---|---|---|---|---|
| | | *mecA* (FAM) | *vanA-vanB* (HEX) | *mecA* (FAM) and *vanA-vanB* (HEX) | | | | | | | | |
| ATCC BAA-2094 MRSA (*mecA* positive) | Positive control | Positive (15.02±0.14) | Negative (N/A) | Positive (FAM, 15.96±0.06) | *mecA* primer | KT878319.1 | *Staphylococcus aureus* strain MSTP6 penicillin binding protein 2a (mecA) gene, partial cds | 278 | 278 | 100 % | 3e-70 | 99.35 % |
| NCCP11517 VRE (*vanA* positive) | Positive control | Negative (N/A) | Positive (16.07±0.02) | Positive (HEX, 15.88±0.05) | *vanA-vanB* primer | OR251469.1 | *Enterococcus faecium* strain 732558 plasmid pEfm732558-vanA, complete sequence | 316 | 316 | 97 % | 9e-82 | 98.88 % |
| NCCP11519 VRE (*vanB* positive) | Positive control | Negative (N/A) | Positive (15.45±0.06) | Positive (HEX, 15.41±0.04) | *vanA-vanB* primer | KT003982.1 | *Enterococcus faecium* strain 08_3270 VanY, VanW, VanH, VanB, and VanX genes, complete cds | 318 | 318 | 98 % | 2e-82 | 99.43 % |
| ATCC29213 *Staphylococcus aureus* | Negative control | Negative (N/A) | Negative (N/A) | Negative (N/A) | - | - | - | - | - | - | - | - |

| | | | | | | | | | | | | |
|---|---|---|---|---|---|---|---|---|---|---|---|---|
| *(mecA negative)* | | | | | | | | | | | | |
| KCTC3511 *Enterococcus faecalis* *(vanA and vanB negative)* | Negative control | Negative (N/A) | Negative (N/A) | Negative (N/A) | - | - | - | - | - | - | - | - |

Abbreviations: NCCP, National Culture Collection for Pathogens, Cheongju, Chungcheongbuk-do, South Korea; KCTC, Korean Collection for Type Cultures, Taejon, Korea; ATCC, American Type Culture Collection, Rockville, MD, USA; MRSA, methicillin-resistant *Staphylococcus aureus*; VRE, vancomycin-resistant enterococci; SD, standard deviation; N/A, not detected.

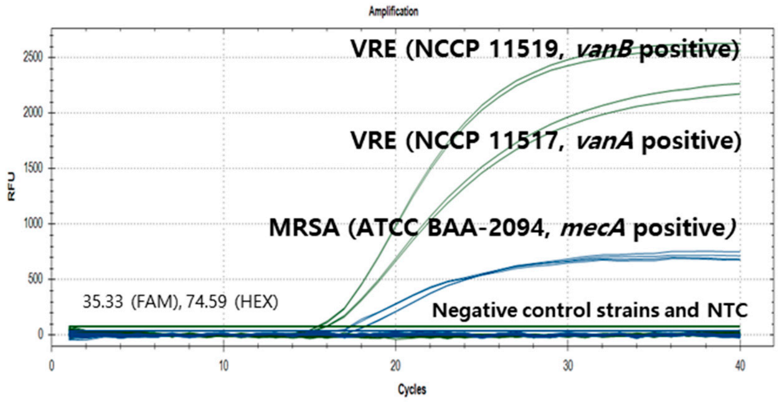


**Figure S1.** Amplification curve positive control strains, negative control strains, and NTC. To confirm the specificity of the assay for *mecA* and *vanA*-*vanB* genes, the reference strains carrying *mecA*, *vanA*, and *vanB* were used as positive controls, including ATCC BAA-2094 *S. aureus* (*mecA* positive), NCCP 11517 *E. faecium* (*vanA* positive), and NCCP 11519 *E. faecium* (*vanB* positive). Meanwhile, the negative controls included methicillin-susceptible *S. aureus* (MSSA) ATCC 29213 and vancomycin-susceptible *E. faecalis* (VSE) KCTC 3511. Multiplex hydrolysis-based real-time PCR was performed for *mecA* and *vanA*-*vanB* genes, respectively. This multiplex real-time qPCR assay amplified the three reference strains of the positive controls, but did not amplify the two reference strains of the negative controls, and no cross-reactivity was found. (Abbreviations: MRSA, methicillin-resistant *Staphylococcus aureus*; VRE, vancomycin-resistant enterococci)
